# Advances in Selective Laser Melting of Nitinol Shape Memory Alloy Part Production

**DOI:** 10.3390/ma12050809

**Published:** 2019-03-08

**Authors:** Josiah Cherian Chekotu, Robert Groarke, Kevin O’Toole, Dermot Brabazon

**Affiliations:** 1 School of Mechanical & Manufacturing Engineering, Dublin City University, Dublin 9, Ireland; robert.groarke@dcu.ie (R.G.); dermot.brabazon@dcu.ie (D.B.); 2 Advanced Processing Technology Research Centre, APT, Dublin City University, Dublin 9, Ireland; 3 I-Form Advanced Manufacturing Research Centre, Dublin City University, Dublin 9, Ireland; 4 Exergyn, DCU Alpha, Old Finglas Road, Glasnevin, Dublin 11, Ireland; kevin.otoole@exergyn.com

**Keywords:** Nitinol, shape memory, superelastic, phase transformation, operation parameters, microstructure, heat treatment

## Abstract

Nitinol (nickel-titanium or Ni-Ti) is the most utilized shape memory alloy due to its good superelasticity, shape memory effect, low stiffness, damping, biocompatibility, and corrosion resistance. Various material characteristics, such as sensitivity to composition and production thermal gradients, make conventional methods ineffective for the manufacture of high quality complex Nitinol components. These issues can be resolved by modern additive manufacturing (AM) methods which can produce net or near-net shape parts with highly precise and complex Nitinol structures. Compared to Laser Engineered Net Shape (LENS), Selective Laser Melting (SLM) has the benefit of more easily creating a high quality local inert atmosphere which protects chemically-reactive Nitinol powders to a higher degree. In this paper, the most recent publications related to the SLM processing of Nitinol are reviewed to identify the various influential factors involved and process-related issues. It is reported how powder quality and material composition have a significant effect on the produced microstructures and phase transformations. The effect of heat treatments after SLM fabrication on the functional and mechanical properties are noted. Optimization of several operating parameters were found to be critical in fabricating Nitinol parts of high density. The importance of processing parameters and related thermal cooling gradient which are crucial for obtaining the correct phase structure for shape memory capabilities are also presented. The paper concludes by presenting the significant findings and areas of prospective future research in relation to the SLM processing of Nitinol.

## 1. Introduction

Shape memory is a unique property of certain metallic and polymeric materials by which they can recover their primary shape (programmed shape) after deformation (under temperature or stress conditions) when a thermal or mechanical force is applied. This ability of shape-memory materials enables them to be used as functional materials in various engineering applications, such as in sensors and actuators, smart structures, biomedical implants and aerospace components [1,2]. Nitinol (nickel-titanium alloy) is the most utilized shape memory alloy (SMA) with other common alloys being CuZnAl and CuAlNi. Unlike most intermetallics, which are brittle, Ni-Ti is ductile in nature, and therefore, often preferred [1,3]. Apart from shape memory properties, Ni-Ti exhibits other desirable characteristics such as good biocompatibility, low stiffness, good corrosion resistance, high wear resistance, high ductility, superelasticity and strength properties. These properties make Nitinol a high potential candidate material for use in medical, aerospace, automotive and heat engine applications. Various commonly used compositions of Nitinol powders were of Ni(45%)-Ti(55%); Ni(50%)-Ti(50%); Ni(55%)-Ti(45%), and their variations. The material has low anisotropy and small grain size compared to other alloys [1,4,5]. Nitinol has a unique set of functional properties based on a reversible martensitic phase transformation. These include shape memory effect, pseudoplasticity (thermal behavior), and pseudoelasticity (mechanical behavior). Most applications of Nitinol in the medical field rely on the superelastic property, whereas the shape memory effect is used in actuators and heat engine applications. These behaviors primarily depend on the transformation temperatures, which vary with the percentage composition of Ni and Ti. A higher content of Ti results in higher transformation temperatures and in a prominent shape memory effect, whereas a higher Ni content lowers the transformation temperatures and bestows superelastic properties upon the material [2,3].

Nitinol alloys are difficult to fabricate and process because of the high spontaneity of titanium, and very low machinability. Conventional processing methods, including casting and powder metallurgy, have several challenges in the processing of Nitinol. These challenges can be summarized as follows [1,3,6,7].

- Achieving a uniform and homogeneous composition
- Producing complex geometries
- Low level of machinability (high elastic and abrasive nature of Ni-Ti)
- Purchasing of good Ni-Ti powder quality (free of oxides and inclusions)
- Provision of inert atmosphere (to avoid oxidation)
- Avoiding impurities (affects transformation temperatures, and crack propagation)
- Avoiding flaws and undesired porosity (reduces load, initiates crack nucleation/propagation)

### 1.1. Additive Manufacturing of Nitinol

Additive manufacturing (AM) was found to be effective in creating highly complex Ni-Ti geometries with pre-designed porosity, homogeneous composition, and desirable properties as compared to the traditional techniques. The process can achieve structures with high density and near net shape, requiring very little or no post-processing. Laser-based AM techniques are progressively being applied to produce Nitinol parts (bulk and porous structures) [1]. For instance, processing Nitinol for bone implants is much easier through AM techniques compared to the difficulty of machining such components with conventional processing methods. AM process parameters can be adjusted to create comparable properties to those of conventionally processed Nitinol, including surface morphology and shape memory characteristics [8]. Among the various additive manufacturing techniques, Laser Engineered Net Shaping (LENS) and Selective Laser Melting (SLM) are the most utilized techniques [9]. The SLM process (also known as Powder Bed Fusion) starts with spreading a thin layer (thickness < 100 µm) of Nitinol powder on a substrate. A high-power computer-controlled laser beam is then used to scan the powder bed. The powder particles melt by absorbing the energy from the laser beam, and solidify to form a cross-sectional layer of the input CAD model slice. This cycle is repeated layer-by-layer with the help of a spreader/recoater blade and adjustable build platform, until the complete part is built [10]. In LENS (also known as Direct Energy Deposition), a solid-state laser beam is focused on the building platform and used to melt the coaxially sprayed metal powder which solidifies in place [11]. Compared to conventional methods, both of these techniques have the potential to provide more gradual phase transformations through produced parts. Both LENS and SLM can fabricate Ni-Ti with complete shape memory recovery for about 6% microscale and 3% macroscale strains. The strains are stabilized at 4% and 2% after cycling [12,13]. However, the SLM process exhibits better processing of Nitinol over the LENS method [12,13], and therefore, it is preferred. A brief comparison between both of these methods is tabulated in Table 1.

### 1.2. Current Work

SLM processing is emerging as one of the most effective ways to produce Nitinol with desirable functional properties. However, relatively little research work has been conducted on this topic. The current paper identifies the advances and progress in the SLM processing of Nitinol. It summarizes various factors such as process parameters, powder characteristics, and heat treatment conditions, and identifies their effect on the microstructures, mechanical characteristics and transformation properties of SLM-fabricated Nitinol samples. A later section also describes the various difficulties associated with producing retained shape memory properties, and several frequent defects and their causes. This study is of high relevance to researchers and engineers who work in this area, to assist them in optimizing the SLM fabrication process for producing Nitinol for specific applications. As this is an emerging area of research, future directions for research work are identified in the concluding section.

## 2. Materials and Methods

### 2.1. Effect of Operation Parameters

In the additive manufacturing of Ni-Ti, processing parameters have the biggest influence on the final product. This product may require higher levels of post-processing, depending on the quality/design requirements. Optimization of these process parameters is, therefore, of real significance. The various process parameters include laser-related, scan-related, powder, and environment (temperature and oxygen concentration)-related factors.

#### 2.1.1. Process Parameters

Energy density has been found to have a directly proportional relationship with strength and impurity levels of SLM fabricated Nitinol components. Higher levels of energy density can improve the density of SLM-fabricated Nitinol products. However, there could be a rise in impurity pick-ups. The relation is as given below, where *E* is energy density, *P* is laser power, *v* is scan speed, *h* is hatch spacing, and *t* is layer thickness [14].

(1) $$E=\frac{P}{v.h.t}$$

Even though SLM fabrication is usually conducted in an inert environment, oxygen and nitrogen are picked up significantly (about 0.14 wt.%) when the energy density is increased [15]. An energy density of 100–200 J/mm^3^ is advised, and a value of 195 J/mm^3^ is recommended for Ni-Ti fabrication using the SLM technique [15]. Walker et al. [6] demonstrated that very high energy density can form wavy surfaces on parts. Very low energy density was found to cause discontinuous melts. Increasing the energy density beyond a critical value was also found to induce porosity, which led to a drop in relative density [16]. High energy density increases the molten pool volume, hindering the escape of gas bubbles to the surface during solidification, and this often results in pores. Secondly, a balling effect can occur at high energy, and may form voids [13].

The laser scan speeds are found to have a direct effect on the thermal hysteresis of the alloy. The functional properties are linked to the thermal hysteresis. Variations in cooling conditions may result in tiny localized variations in chemical composition [9,17]. During the SLM process, the heat distribution is of a non-uniform nature. Due to this, certain regions are exposed to more heat, and Ni evaporation may occur, forming Ti_2_Ni precipitates [13]. It was also noted that lower scan speeds can exhibit higher thermal memory recovery, and improved superelasticity [18]. Other intermetallic phases such as Ti_3_Ni_3_ and TiNi_3_ may precipitate, due to a loss of laser control over the synthesis (e.g., during exothermal reaction) for a certain period [19]. Relatively low scan speeds (<200 mm/s) or high energy densities may create intermetallic phases such as Ti_2_Ni, Ti_3_Ni_4_, or TiO_2_ and Ti_4_Ni_2_O oxides, and result in poor phase transformations affecting the functional properties [20].

If the operation environment has a high oxygen content (≥1800 ppm), brittle oxides may form and exhibit undesirable mechanical responses [13]. Non-metallic elements such as oxygen and carbon are often picked up during the sintering process. Ni-Ti is often sensitive to impurity pick up while processing at high-temperatures [21,22]. This results in impurity-related brittle Ti-rich phases such as Ti_4_Ni_2_O_x_ which could strongly alter the microstructural properties, and thereby, the functional properties [23,24,25]. Another factor to consider in the SLM fabrication process is substrate pre-heating. If the substrate is not heated prior to the sintering process, high residual stresses may appear at the bottom region of the component. This is due to the high thermal gradient between the first few layers and the building platform, and if not attended to properly, may result in a warping effect, causing the fabricated part to separate from the substrate material. Preheating the substrate will decrease the thermal gradient between the first few layers and the substrate [26,27,28].

Optimization of laser parameters is necessary to ensure high density levels and low impurity concentrations in the fabricated Nitinol [15,29,30,31]. According to ASTM F2063-05 [32], the impurity levels must not exceed 500 ppm. The optimum laser parameters can be identified by creating single Nitinol tracks, and modify the parameters until the fabricated tracks meet the desired requirements [29]. A set of optimal process parameters were identified by Walker et al. [6] for SLM processing of NiTi (Phoenix-PXM machine). The parametric values were: laser power = 250 W, scan velocity = 1.25 m/s, spot diameter = 30 µm, and hatch spacing = 120 µm. They were able to develop a relative part density of 98%, and shape memory functionality. Another set of parameters using the same system were suggested by Shishkovsky et al. [19] as: laser power = 50 W, scan velocity = 0.1–0.16 m/s, spot diameter = 70 µm, and hatch spacing = 100 µm. the process obtained 97% relative density. Haberland et al. [15] also used an energy density of 200 J/mm (54.7 J/mm^3^) and produced a fully dense Ni-Ti containing what were considered permissible levels of impurity (O_2_: 0.03–0.04 wt.%; N_2_: 0.01–0.02 wt.%; C: 0.028–0.03 wt.%). The authors also modified the existing energy density relation as follows for simple geometries, to include other parameters such as *d_b_*—beam diameter (mm): *d_t_*—track width (mm): and ρ*_r_*—powder-bed relative density.

(2) $$E=\frac{P}{\rho_{r}\cdot d_{b}\cdot \;t\cdot \;v}\;d_{t}\leq h$$

(3) $$E=\frac{P}{\rho_{r}\cdot d_{b}\cdot t\cdot v}\cdot \left(2-\frac{h}{d_{t}}\right)\;0<h<d_{t}$$

Dadbakhsh et al. [17] studied the difference in phase formation by producing samples at low laser parameters (LP) and high laser parameters (HP), separately under similar energy densities. The LP parameters were associated with a low power (40 W), and low scanning speed (160 mm/s), with low heating and cooling rates. The HP parameters were based on high power (250 W), and high scanning speed (1100 mm/s), with higher heating and cooling rates. The two combinations produced similar densities (99%) and chemical compositions. The fabricated samples via LP conditions exhibited martensitic phase with functioning shape memory effect, while the HP combination produced austenitic phase with a superelasticity property, at room temperature.

In order to retain proper densification levels, a coordinated increment or decrement of the scan speed and laser power was noted as being necessary. It was found that the energy density decreased when laser power was kept constant and scanning speed was increased. Similarly, the energy density decreased when the hatch spacing was increased, while keeping laser power and scanning speed constant [3].

#### 2.1.2. Phases and Crystal Structures of Nitinol

**Figure 1:**
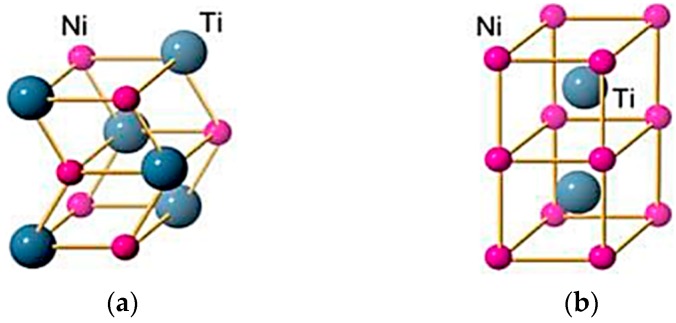
Crystal structure phases of NiTi showing (**a**) B19′ martensite, and (**b**) B2 austenite [3].

There are three different functioning phases in Nitinol. In the martensitic phase, Nitinol has a low symmetry, and complex-twinned monoclinic B19′ structure (Figure 1a). In the austenitic phase, Nitinol has a highly symmetric, and ordered body centered cubic (BCC) crystal lattice structure, denoted as B2 structure (Figure 1b). Martensite is characterized by needle-like crystals arrayed in a herringbone shape. The austenite phase is hard and stiff, while that of martensite is softer, more ductile, and has a lower yield stress. In some grades of Ni-Ti, an intermediate R-phase may be present which has a rhombohedral structure exhibiting low transformation strain, and low temperature hysteresis (1–10 °C) [33,34]. The R-phase formation can be linked to any previous cold working or aging of Ni-rich alloys, or may be due to alloying with an additional element like iron [3].

**Figure 2:**
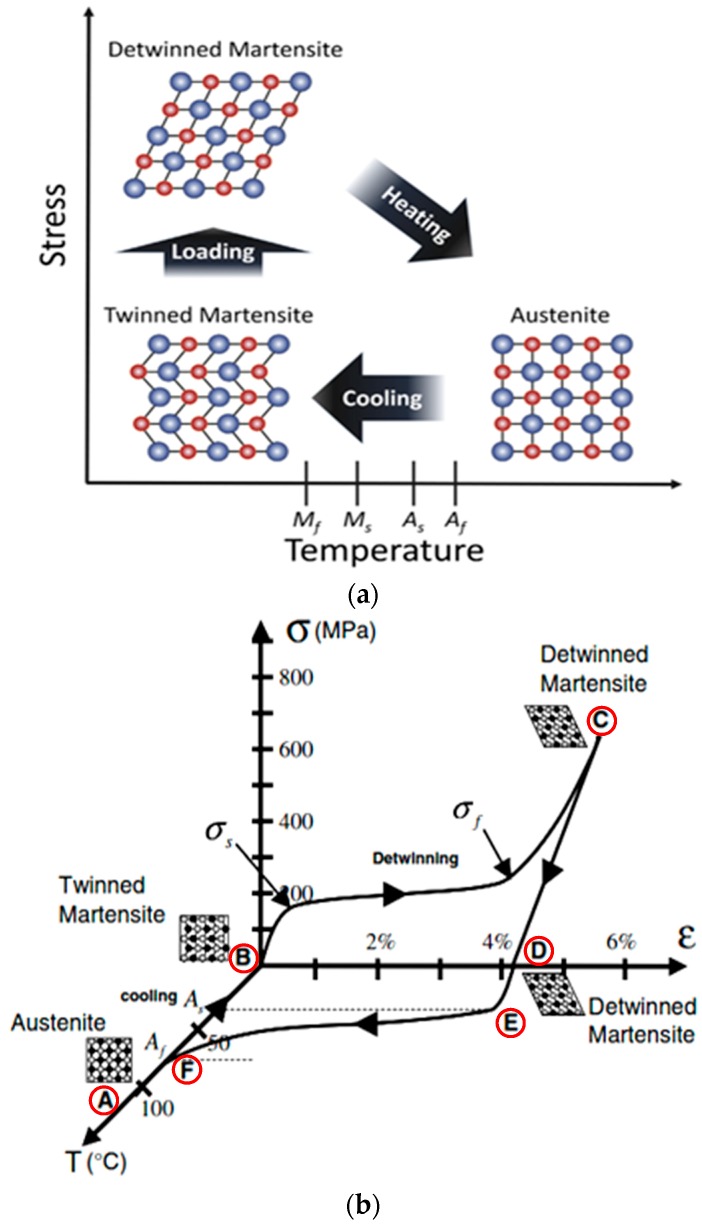
(**a**) Representation of phase transformation in shape memory Ni-Ti [40], and (**b**) Stress-Strain-Temperature graph showing phase transformation in Ni-Ti [41].

**Figure 3:**
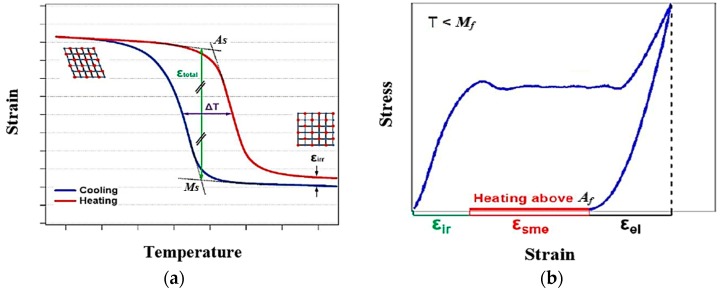
Repeatable cycling of Ni-Ti are shown as (**a**) the thermal (strain-temperature) and (**b**) stress-strain cycles [3].

When Nitinol is heated, and temperature exceeds the transformation temperatures, the martensite transforms into austenite, recovering the original programmed shape. Hence, this shape memory effect is also referred to as the “thermal memory effect” [1]. The schematic representation of this transformation is shown in Figure 2a. A more detailed explanation can be provided based on the different transition temperatures involved in the phase formations (Figure 2b). At room temperature, the material will be in the twinned martensite phase (B). When a deformation is applied, the phase changes to detwinned martensite (C) by reorienting and detwinning the lattice structure. The twin boundaries in martensite shift such that they orient in one preferential direction to better accommodate the load; this phenomenon is termed “detwinning”. This microstructural process enables Ni-Ti to withstand high strain without any permanent deformations [35]. When this detwinned martensite (D) is heated to exceed the austenite start temperature (*As*), austenite begins to form (E), and once it crosses austenite finish temperature (*Af*), austenite formation will be complete (A, F). Figure 3 shows the typical strain-temperature and stress-strain curves of Ni-Ti.

As noted, for some grades of Nitinol, the intermediate phase called the R-phase may form. As the cubic austenite phase is cooled, one of the lattice diagonals elongates resulting at a reduced angle (<90°). Hence, the name R-phase due to the rhombohedral structure it forms. If the material is cooled below the critical R-phase temperature (*Rs*), R-phase crystals may form. The resulting microstructure will contain both austenite and R-phase, and is referred as the pre-martensite phase [36]. When the material is further cooled down to the martensite start temperature (*Ms*), a martensite phase starts to form. The austenite (low strain phase) to martensite (high strain phase) transformation will be completed once it cools down below the martensite finish temperature (*Mf*). Conversely, when the austenite phase is sufficiently stressed, it changes to martensite. The Clausius–Clapeyron stress-temperature relationship for Ni-Ti describes the activation process under stress of the forward transformation from austenite to martensite, as well as the reverse transformation (martensite to austenite). This relationship indicates that the activation temperatures (*As*, *Af*, *Ms* and *Mf*) increase linearly per unit stress [37,38,39]. At higher temperatures, martensite is unstable, and therefore, returns to the austenite phase on unloading. This large elastic response of reversing the deformation to original shape is called superelasticity [1]. This martensitic transformation is a diffusionless shear (solid-state) transformation. A coordinated motion of a large number of atoms relative to their neighbors causes this diffusionless displaced transition. A new crystal structure is formed from parent phase without any change in the composition [3].

#### 2.1.3. Metal AM Parameter Setting Effect on Microstructure

Microstructures with different grain morphology, size, and texture can be tailored by adjusting the process parameters [16,42,43]. The layer-by-layer melting and consolidation creates complex thermal gradients, spatially varying grains, and precipitates in the as-fabricated structure. Complex thermal gradients can occur due to high laser power and cooling rates, resulting in a solidification imbalance [44]. The high cooling rates in the range of 10^3^–10^8^ K/s may form finer powders, improving the mechanical properties and density of the final part [45,46]. Khoo et al. [47] used repetitive laser scanning technique, in which the second scan imparts lesser energy to the powder compared to a single scan method. Thus, the molten pool acquired a lower temperature and a shorter solidification time. Various SLM research papers have confirmed that grains generally orient in the path of the highest thermal gradient (vertical). Grains may display columnar and equiaxed morphologies with varying sizes [16,48,49,50]. The thermal stresses during the SLM processing of Ni-Ti can generate stress-induced martensitic phases. In SLM, the laser power and scanning velocity affects the microstructure distinctly. Even though not much impact on microstructure were reported, change in scan velocities affected the Ni evaporation, altering the phase transformation temperatures [16]. It was further understood that the power density, and complex thermal process during solidification also affects the microstructure.

**Figure 4:**
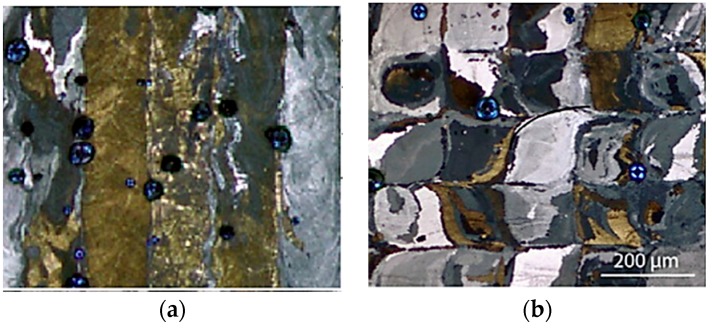
(**a**) Elongated grains as seen in an optical image of SLM-fabricated Ni-Ti (grid columnar style); and (**b**) formation of S-shaped grains due to the laser scanning motion [16].

**Figure 5:**
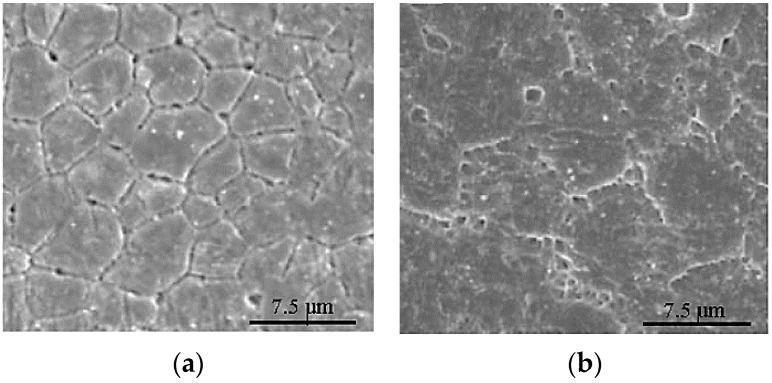
SEM images showing an increase in grain sizes when the laser power is increased (constant scan speed), (**a**) P = 300 W (**b**) P = 500 W [53].

The melting process in SLM involves the re-melting of the formerly sintered layer with bonding of the new layer in an epitaxial solidification manner. Epitaxial solidification is necessary to bond the interlayers strongly, and to prevent undesirable intermetallic phases and porosities in these regions [51,52]. This can result in grain growth in the sublayer, while the current layer grains are sintered [16,44]. Epitaxial growth can result in an increased length and width of the grains [16]. This elongation may be in the form of platelet shape oriented parallel to the laser beam direction (Figure 4), following the higher thermal gradient track (scanning direction). A minimum critical energy level is necessary to maintain this epitaxial solidification and a lower cooling rate. The lower cooling rates can lead to the formation of coarser grains [45,53,54]. Grain sizes increase with increasing laser power (Figure 5) [16,53,55]. In an experiment conducted by Bormann et al. [16], grain shapes were observed to change from s-shape to rectangular when a high laser power (56–100 W) was used [16]. These grains were also found to orient themselves along the heat direction. Lower levels of cooling rate caused re-orientation and enlargement of the formed grains [45]. High laser power may also increase the porosity. High energy input (>74 J/mm) is likely to increase the surface roughness and porosity between the adjacent scan tracks. During prolonged melting or high energy, the molten pool becomes unstable, resulting in irregular tracks and vaporization [17,18,56]. When the input energy is less than a critical level (no epitaxial solidification), the sublayer will not be adequately sintered; this could result in reduced area of contact between the layers. The surface tension causes the formation of a cylindrical shape, higher porosity, and weak bonding between layers. Lower cooling rates are often seen in this case, as are re-orientation and enlargement of the formed grains [57].

#### 2.1.4. Effect on Transformation Temperatures

Dadbakhsh et al. [17] reported that the SLM parameters can highly influence the transformation temperatures and mechanical response of porous and dense Ni-Ti. In the case of SLM processing of Nitinol, it was observed that the phase transformation temperatures increase as the energy increases from 45 to 545 J/mm^3^ [15,42,58]. This effect is due to the evaporation which occurs during laser processing at high energy. Nickel, which has a lower evaporation temperature (2913 °C), evaporates more readily, leaving more titanium (with an evaporation temperature of 3287 °C) in the matrix composition. This higher titanium content increases the transformation temperature [3,11]. High energy density can also evaporate nickel ions (explained earlier), resulting in higher transformation temperatures [59]. This phenomenon is observable in all the compositions i.e., high Ti content or Ni content, or near equiatomic Ni-Ti [60].

#### 2.1.5. Effect on Corrosion Properties

The corrosion properties of Nitinol are of concern from a cytocompatibility viewpoint. Release of Ni ions from the matrix can cause harmful and adverse effects in biological applications. Nitinol is known for its good corrosion resistance, and hence its biocompatibility, due to the rapid development of protective oxide layers (thickness of 2–20 nm) on the surface. The oxides that usually form on the Ni-Ti surface are mostly TiO_2_, along with small traces of TiO, Ti_2_O_3_, NiO and Ni_2_O_3_ [25,61]. However, Ni ions can still leach in physiological environments when exposed to long service durations [62]. Laser-based processing can lead to higher Ti concentration on the surface, and a thicker oxide layer [61]. Alternatively, controlling the Ti/Ni ratio can also result in higher corrosion resistance due to better stability of the TiO_2_ film [63].

In a study performed by [25], both SLM-fabricated and conventionally-produced Ni-Ti was found to exhibit comparable corrosion properties. The capability of SLM in producing a homogeneous and defect-free Ni-Ti structure could further improve the corrosion resistance during service. It was also found that when the porosity was increased, a higher corrosion current was caused due to the presence of larger surface area and an increased number of edges of the porous structures. The corrosion current was observed to increase from about 200 nA (bulk structure) to about 950 nA (35% porous structure). Higher corrosion currents could result in a higher release rate of Ni ion and corrosion by-products [25]. If the operation environment contains higher oxygen content (e.g., 0.6%), the SLM process can form large oxide film on the porous structure. This can affect the mechanical property; however, it will increase the corrosion resistance and biocompatibility [64,65]. As mentioned in the previous section, low cooling rates can create coarser grains, thus reducing the extent of grain boundaries, which are potential nucleation sites for precipitates or impurities. Therefore, controlling the cooling rates during SLM can directly translate into a reduction in corrosion rates for Ni-Ti [64,66,67,68].

### 2.2. Powder and Material Composition

#### 2.2.1. Effect on Microstructure

**Figure 6:**
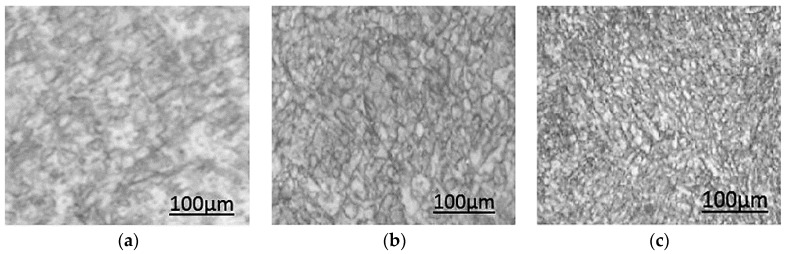
Different grain characters as seen in the microstructural images of (**a**) Ni45Ti, (**b**) Ni55Ti, and (**c**) Ni50Ti [71].

Powder shape and particle size distribution are also found to affect the microstructure [69,70]. Laser processing often changes the microstructures and phases of the feedstock alloy powder. Due to the lower heat flux near supports, the grains usually become finer in the regions far away from the base, while coarser grains are formed at the bottom region of the fabricated component [45,54]. In a study conducted by Shiva et al. [71], it was observed that equiatomic Ni(50%)-Ti(50%) composition presented the most uniform, finest, and highest packed grains compared to the other two compositions. Ni(45%)-Ti(55%) exhibited uniformly distributed grains of irregular shapes and large sizes. Ni(55%)-Ti(45%) exhibited smaller grains when compared to Ni(45%)-Ti(55%); however it was still large when compared to Ni(50%)-Ti(50%) composition (Figure 6). Finer particles are preferred for denser fabrication, and they also reduce the energy requirement for epitaxial solidification [45]. Particle sizes in the range of 20–63 μm or smaller exhibited poor flowability, and a reduced packing density (56%). When the range was around 25–75 μm and 45–100 μm, a higher packing density (60%) was observed. If the particle size falls below 45 μm, packaging density was about 44%; below 25 μm, the packing density fell even further to about 37% [15,49]. For SLM of Ni-Ti, medium-sized fractions around 25–75 µm are the ones with favorable particle size, spherical morphology, flowability and packing density, impurity content, and excellent transformation ability.

#### 2.2.2. Effect on Transformation Temperatures

**Figure 7:**
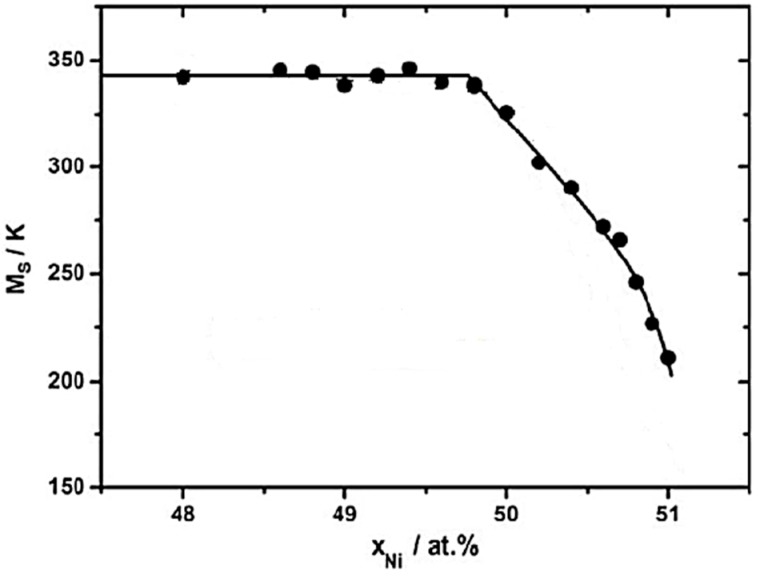
Influence of nickel content on martensite start temperature [75].

**Figure 8:**
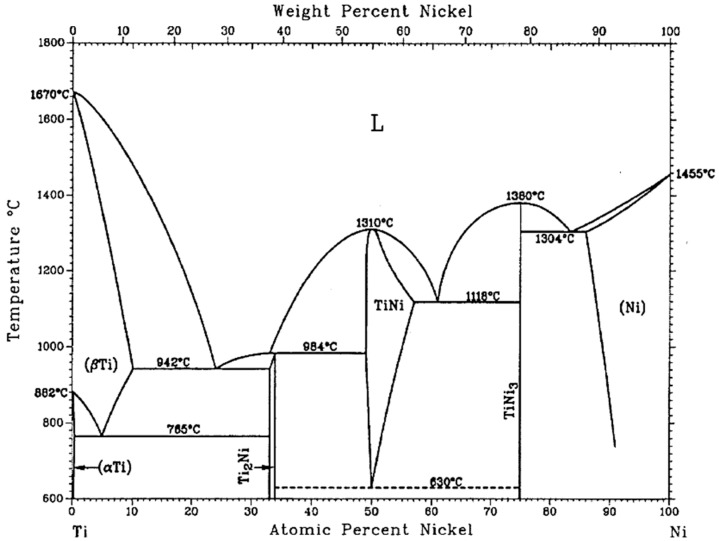
Binary phase diagram of Nitinol (Ni-Ti) alloy [2].

Transformation temperatures of Nitinol are very susceptible to the Ni or Ti content. Near equiatomic Ni-Ti have disadvantages such as lower strength and poor cyclic stability compared to a Ni-rich Nitinol. Higher titanium content in the matrix composition will need higher temperature to process, owing to the higher melting point of Ti. Binary Ni-Ti alloys have transformation temperatures between −40 °C and 100 °C and exhibit a temperature hysteresis of 20–40 °C. Higher Ni content (lower Ti content) can decrease the transformation temperatures at a rate of about 93 °C/at.% Ni content. For instance, the effect of nickel content on martensite start (*M_S_*) temperature is shown in Figure 7. As nickel content increases, *M_S_* temperature decreases [3,6]. It has been reported that 50Ni and 50.5Ni (at.%) Nitinol failed to show pseudoelasticity due to low strength [3]. The diffusionless and reversible martensite-austenite transformation takes place in the temperature range of 50–100 °C as a function of the nickel content. This is associated with a variation of transformation temperatures by approximately 10 °C/0.1 at.% change in the nickel content [72,73]. Increasing nickel content also increases the critical stress needed for martensitic transformation and the strain recovery. Therefore, the chemical composition should be maintained very accurately. The transformation temperatures of Ni-Ti alloys are also very sensitive to impurities such as nitrogen, carbon, and oxygen [2]. For instance, if oxygen is present in the Nitinol matrix, the transformation temperature will be lowered, and causes the parent phase to be brittle. Studying the phase diagram (Figure 8), we can see the existence of few stable phases (Ni_3_Ti, NiTi_2_) besides the main phase Ni-Ti. These additional phases will not exhibit shape memory property and their presence affect the composition of the remnant Ni-Ti portion of matrix. This will also affect the transformation temperature. A metastable Ni_4_Ti_3_ phase precipitates at lower temperatures owing to the decreased solubility of nickel. This phase is coarsened when annealed at 300–600 °C, resulting in the formation of a stable Ni_3_Ti phase [2]. Samples lacking these precipitates (high homogeneity) are highly suitable for medical applications. The Ni^+^ ion release can be reduced by using a smaller laser spot size, lowering risks of Nitinol biomedical implants [13,74].

### 2.3. Heat Treatment Processes

#### 2.3.1. Effect on Phase Transformations

**Figure 9:**
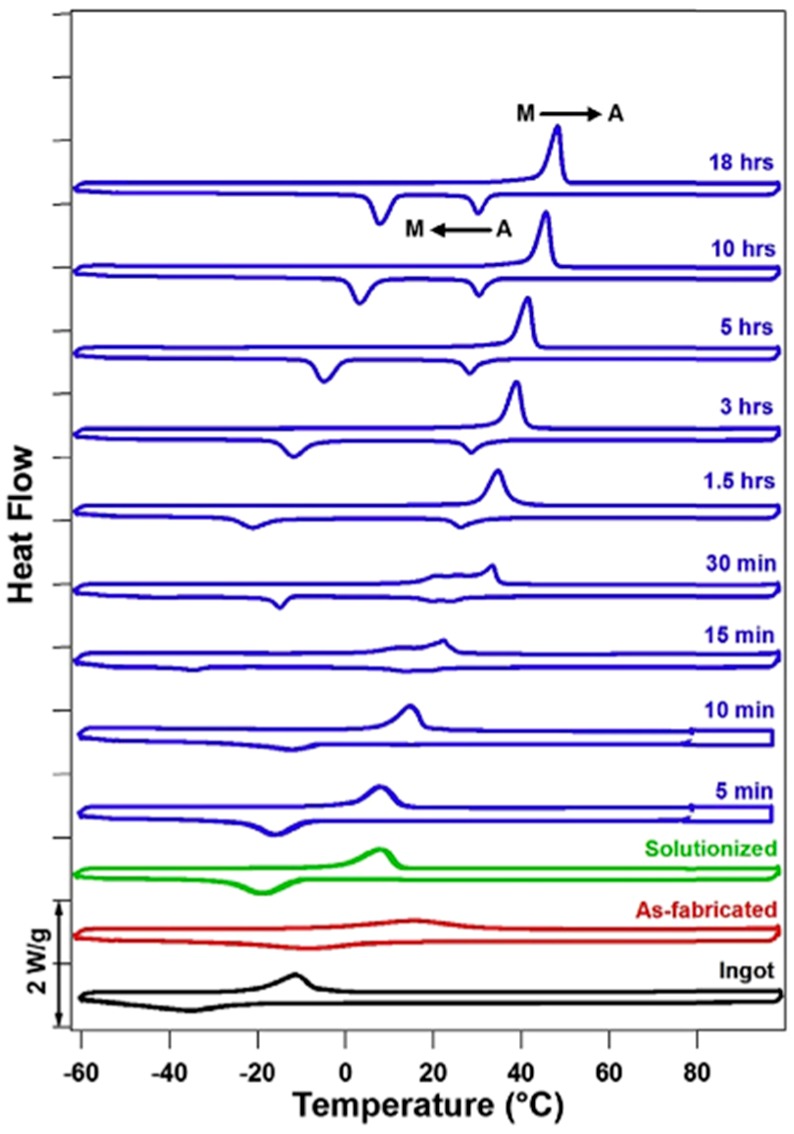
Differential Scanning Calorimetry plot showing the effect of solution annealing and ageing time (blue curves) on Ni(50.8 at.%)Ti Nitinol [78].

The as-fabricated Ni-rich Nitinol will not be able to recover the full strength because of the nucleation of martensite in an austenite region [76]. Heat treatments such as solution annealing and aging can be applied after SLM fabrication to provide a homogeneous equilibrium state throughout the material, and help in recovering the strength. It also helps in clearing several microstructural defects and residual stresses [77]. Subsequent aging processes can be applied in order to recover strength completely through precipitating Ni-rich phases such as Ni_3_Ti, Ni_3_Ti_2_, and Ni_4_Ti_3_. Solution annealing followed by water quenching could cause significant decrease in transformation temperatures of Ni-rich Nitinol. The metastable phases such as Ni_3_Ti_2_ dissolve during solution annealing and this suppresses further precipitation when water quenching is employed. Solution annealing also decreases the transformation features such as peak width (in Differential Scanning Calorimetry). This was confirmed by Andani et al. [58] and Saedi et al. [78] who reported lower transformation temperatures (about 20 °C) and a single-phase transformation. It was also found that a longer aging duration may increase the transformation temperatures (Figure 9). This could be either due to evaporation of Ni in prolonged high temperature conditions, or precipitation of Ni-rich phases at high temperatures [78,79]. Oxidation happens often during the heat treatment processes at high temperatures. This may result in the reduction of Ti, as it is highly reactive to oxygen, and consequently, it decreases the transformation temperatures. This could further result in poor martensite to austenite transformations and an elevation in precipitate formation [80].

When the martensite transformation is suppressed by a solution treatment and a subsequent ageing processes, formation of R-phase could occur. This is due to the introduction of Ni-Ti precipitates (usually Ni_4_Ti_3_) which could favor the R-phase growth [36,47]. In the DSC plots, we may see distinct peaks; the first peak indicates the austenite to R-phase transformation, while the second represents the transformation from R-phase to martensite phase [15,81].

#### 2.3.2. Effect on Mechanical Properties

The thermal stresses during SLM can generate stress-induced martensitic phases. Solution annealing can homogenize the composition, dissolve all precipitates, and eliminate all stress-induced phases [1,78]. Saedi et al. [78] conducted a Vicker’s hardness tests on SLM fabricated Ni-Ti sample, and the hardness value was found to be 224 HV, which is much lower than the ingot hardness (278 HV). Employing solution annealing increased the hardness value to 288 HV. Solution annealing dissolves the brittle Ni_4_Ti_3_ precipitates; the increase in the hardness was ascribed to this. A sample which is subjected to an aging process for 18 h at 350 °C. Following this, annealing can eventually exhibit an increase in hardness up to 345 HV. Haberland et al. [82] used a Ni-rich specimens to study the effect of orientation on the compressive properties of superelastic Ni-Ti. Their samples were solution annealed at 950 °C for 5.5 h after fabrication, and then quenched under water. The heat treatments generate a more flat loading curve (versus steep curve), and greater stress and strain at failure. This was caused by the dissolution of nucleated Ni_4_Ti_3_ phases which had previously formed. Generally, these precipitates hinder plastic deformation. Subsequent aging processes can result in reducing the fracture stress and strain. However, it was found that more severe aging conditions (temperature and duration) create a more ductile curve and higher values of fracture stress and strain [36]. Saedi et al. [78] also observed that the subsequent aging also increased the yield stress by about 700 MPa. These observations were because of the precipitation/age hardening effect.

### 2.4. Challenges in Producing Shape Memory Effect

**Figure 10:**
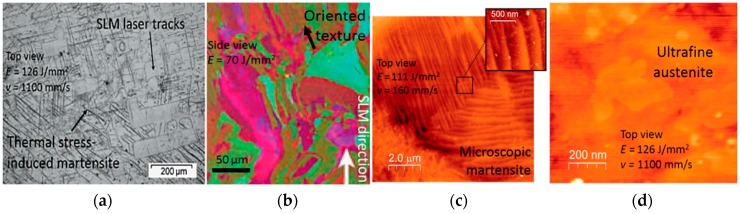
(**a**) SLM laser tracks having stress-induced martensite due to high thermal stresses; (**b**) formation of fine austenite sub-grains along SLM build direction [42]; (**c**) AFM image showing fine internally twinned martensitic structure; (**d**) AFM image showing ultrafine austenite sub-grains [1].

Achieving an effective shape memory property is challenging, owing to high localized heating, high scan speed, fast heating rates, and rapid solidification rates. The shape memory effect and mechanical responses depend significantly on the microstructural characteristics. Apart from operation parameters, the microstructural characteristics can also be affected by laser solidification tracks, the formation of very small austenitic grains inside melt pools, large plate-like martensitic phases that are thermal stress-induced, and a preferential texture corresponding to the heat flow direction. Compared to LENS, these factors are more profound in SLM, as scanning velocities are much higher. Figure 10 shows these occurrences in an SLM-fabricated Ni-Ti. These typical features will eliminate the residual elastic energy among the laser tracks; however, it will reduce the uniformity of martensitic phase transformations. The grains always tend to orient along the build direction (heat flow direction). This can produce large anisotropy in stiffness and shape memory responses. Fine martensitic and austenitic sub-grains formed in laser tracks can also be seen in the Figure 10 [1]. The large martensitic plates can be removed by proper annealing and furnace cooling. However, these treatments will not produce isotropic properties. The application of furnace cooling after annealing can result in the segregation of martensite and austenite within the solidified track, leading to further mixed shape memory behavior [13]. Dadbakhsh et al. [48] reported the influence of orientation of austenitic crystals on shape memory response of Ni-Ti fabricated by SLM. The structures in which the austenitic crystals are aligned vertically showed the highest elastic recovery. Conversely, the horizontal alignment exhibited lowest elastic recovery and highest shape memory recovery strain. This discrepancy is due to the presence of elongated austenitic crystals, which may destabilize the twinned martensite. The horizontal orientation can also increase the material’s resistance to compressive loads [13]. Highly dense SLM-fabricated equiatomic Ni-Ti showed almost full shape memory recovery, while in porous samples, about 0.5% irrecoverable strain remained. However, the superelasticity property shows only partial recovery due to low strength property of equiatomic Ni-Ti. The cycling tests will gradually stabilize the shape memory behavior, and irrecoverable strain becomes negligible [3,82,83].

### 2.5. Defects in Fabricated Parts

High thermal gradients and cooling rates can increase the chances of formation of columnar grain structures. These columnar grains may lead to shrinkage-related defects such as solidification cracks. This can be controlled by changing the alloy composition or decreasing the rate of solidification [84]. The lased-based AM processes (SLM or LENS) are expected to maximize the material density [1]. However, porosity is recognized as one of the most common quality issues. SLM can be used to pre-design pore morphology. By engineering the porosity, a desired stiffness value can thereby be achieved [70].

**Figure 11:**
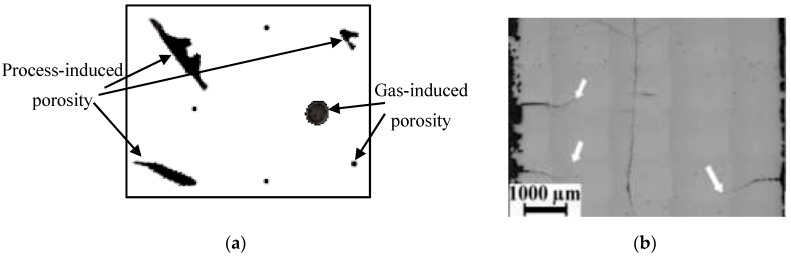
(**a**) Schematic representation of gas-induced and process-induced porosities; (**b**) Optical micrograph showing cracks [93].

Cracks and pores are the two most common structural defects found in SLM-fabricated Ni-Ti [84,85]. The porosities can be classified based on the cause of occurrence; gas-induced or process-induced porosity (Figure 11a). Gas induced pores are caused by trapped gas in the powder bed. Pores can form near the edge regions due to insufficient melting of powder particles, and they are referred to as “process induced pores” [86]. Based on shape morphology, pores can be of spherical and irregular types [44]. Spherical porosity is due to the mixing of ambient gas with the Nitinol powder particles; the trapped gas does not have enough time to escape from the melt pool. Irregular pores are formed due to the balling effect. These pores can be reduced by epitaxial solidification [87]. Strong bonding between layers is necessary to prevent chances of pore formation (optimize laser power and scan speed). It has been reported that energy densities of more than 74 J/mm^3^ would increase chances of pores [9]. Irregular pores can be reduced by using an inert gas (Argon) environment to reduce the oxygen levels during processing. This could also reduce the balling effect (oxygen expand between layers) due to less oxygen, further reducing the chances of irregular pores [57,88,89].

Owing to the process nature, SLM and LENS have a high tendency to create unbalanced stress profiles (residual thermal stresses) between the printed layers. Residual stresses in these processes, usually occur due to the large thermal gradients created by several re-melting and cooling cycles, taking place at inconsistent heat levels or thermal gradient levels. The regions of concern are mainly the exposed layer (top layer) and the interface between the exposed layer and the previously printed layer [90]. Thermal expansion of the top layer creates tensile stress, while the cooler layer below undergoes compressive stresses. This phenomenon occurs throughout the underlying layers, and may affect the same layer multiple times. This will eventually result in a stress gradient between the layers. Residual stresses are usually highest in the scan overlap regions. Residual stresses, if high enough, can initiate cracks (Figure 11b) throughout the Nitinol samples, and reduce the fatigue strength drastically. This can result in the delamination and/or warpage of feature geometry [78,89,90]. Another reason for crack formation could be the balling effect between layers, or element vaporization. Hence, the process parameters should be such that they favor epitaxial solidification, and also provide an inert process atmosphere [87,89,91]. Element vaporization is another cause of crack-initiating pores in the structure. These cracks, however, do not propagate as the SLM-inherent rapid cooling prevents the propagation mechanism [91,92].

## 3. Conclusions

In this paper, previously published work related to the SLM processing of Nitinol has been reviewed to identify and quantify the influential factors involved, process-related issues, and suggest possible areas to work on. Based on the findings to date, it is clear that additive manufacturing could be used to process Nitinol components with high density and near net shape, requiring very little or no post-processing. Out of the two common AM approaches (LENS and SLM) for Nitinol processing, SLM has been reported as the preferred method. The SLM process can be used to produce parts with homogenous and comparable composition to that of the feedstock. The process was also found to be capable of forming uniform microstructure with high aspect ratio columnar grains, uniform strain accumulation and a stable detwinned martensite phase structure.

The SLM process parameters were found to have a significant impact on the microstructure and phase transformation temperatures. The optimization of several parameters including energy density, scan velocities, and working environment was shown to be necessary to attain high density levels and low impurity levels in the fabricated Nitinol parts. Too high or low energy densities result in impurity pickup, porosity, and wavy surface finishes. The scan speeds of the laser beam affect the thermal hysteresis due to longer heat exposure, which in turn influence the shape memory and pseudoelastic properties. The presence of nitrogen, carbon, and oxygen in the work chamber is found to alter the transformation temperatures of Nitinol. A high oxygen content can cause degradation of the mechanical and functional properties in the material. Complex thermal gradients affect the microstructure of the as-fabricated structure. Substrate preheating is recommended to reduce the thermal gradient and relieve the residual stresses. Density levels can be managed through coordinated manipulation of scan speed and laser power. High cooling rates were found to generally improve the mechanical properties and density. However, they may result in large thermal gradients affecting the residual stress, grain orientations and phase formations. To avoid undesirable intermetallic phases and porosities, epitaxial solidification has been found to be effective, which could be achieved by controlling the input energy and cooling rate. Higher energy levels cause evaporation of Ni, and therefore, result in higher phase transformation temperatures, as higher Ti content in the composition will increase the processing as well as the transformation temperatures. From the control of cooling rates and oxygen content in the process environment, it was found to be possible to improve the corrosion protection of Nitinol. The creation of low density (porous) structures can also increase the chance of corrosion occurring.

The powder quality and material composition also impose a significant effect on the microstructure and phase transformations. Equiatomic Ni-Ti was observed to exhibit the most uniform and finest grains, which is highly recommended for high density fabrication. Particle size was also found to affect the flowability and packing density of the feedstock during fabrication. The martensitic transformation and strain recovery are also influenced by the Ni content in the composition, and therefore maintaining an accurate composition is necessary. Heat treatments performed post-SLM are also reported to affect the functional and mechanical properties to a great extent. More ductile curves and high fracture characteristics were created when heat treatments involving very high temperatures and durations were used. High localized temperature rise, scan speeds, heating and solidification rates make it difficult to produce the right phase structure for shape memory effect via SLM process. The method of solution annealing was found to be effective in homogenizing the composition and dissolving and removing the undesired precipitates and the stress-induced phases.

The difficulties in producing effective shape memory response lies mostly in the microstructural characteristics, which may include formed grain morphologies, stress-induced phases, and preferential texture formations. Annealing and furnace cooling treatment can improve the functional properties; however, more prospective work is required to completely eliminate the anomalies leading to mixed functional responses during SLM fabrication of Nitinol parts. Furthermore, the common structural defects have also been reviewed. It was found that pores and cracks are two prominent process-related defects which result during SLM processing. Common causes may include gas trapping, insufficient melting, high thermal gradients and cooling rates.

The research and development on this topic remains at an early stage and significant work will have to be conducted to enable Nitinol parts to be effectively produced via metal AM. More studies are required to be focused on optimizing the parameters to consider dimensional precision, surface quality, and the functional properties response. SLM process parameters need to be better analyzed for optimized control to achieve repeatability in SLM processed Nitinol, and to produce components with superior quality and reliability. Residual stresses, being one of the significant problems, must be examined to find better solutions to mitigate them. Only a few studies have been conducted to better understand the effect of operation parameters in causing porosity and crack defects. The fatigue behavior of SLM-produced Nitinol must be studied further to reduce the numbers of defects, and achieve higher reliability of functional responses. An in-depth understanding is required to interlink the functional and mechanical properties with the microstructural characteristics resulting from a set of process parameters.

## Figures and Tables

**Table 1:** Comparison between Selective Laser Melting (SLM) and Laser Engineered Net Shape (LENS) processing of Nitinol [12,13].

SLM	LENS
• Fabricates homogenous composition equivalent to the composition of the feedstock	• Composition varies spatially and may differ from the powder feedstock composition.
• Exhibits high aspect columnar grains extending over multiple layers, due to orientation in the build direction (along largest thermal gradient).	• Equiaxial grains are created with dimensions corresponding to the layer thickness (has smaller minor axis).
• Strain accumulation is more uniform, as material microstructure is more homogeneous. • The stress-strain curve shows a plateau as per the response when critical stress is exceeded.	• Due to heterogeneity, strain accumulation varies. The stress-strain curve exhibits a strain-hardening like response once the critical stress was crossed.
• Shape recovery requires a temperature increase. • Detwinned martensite is more stable.	• Residual martensite in the fabricated microstructure recovers on heating, hence, detwinned martensite is less stable in LENS-fabricated components.
